# The Role of Neuro-Immune Interaction in Chronic Pain Conditions; Functional Somatic Syndrome, Neurogenic Inflammation, and Peripheral Neuropathy

**DOI:** 10.3390/ijms23158574

**Published:** 2022-08-02

**Authors:** Elaine Meade, Mary Garvey

**Affiliations:** 1 Department of Life Science, Atlantic Technological University, F91 YW50 Sligo, Ireland; elaine.meade@mail.itsligo.ie; 2 Centre for Precision Engineering, Materials and Manufacturing Research (PEM), Atlantic Technological University, F91 YW50 Sligo, Ireland

**Keywords:** chronic pain, neurogenic, immunogenic, functional somatic syndrome, co-morbidities

## Abstract

Functional somatic syndromes are increasingly diagnosed in chronically ill patients presenting with an array of symptoms not attributed to physical ailments. Conditions such as chronic fatigue syndrome, fibromyalgia syndrome, or irritable bowel syndrome are common disorders that belong in this broad category. Such syndromes are characterised by the presence of one or multiple chronic symptoms including widespread musculoskeletal pain, fatigue, sleep disorders, and abdominal pain, amongst other issues. Symptoms are believed to relate to a complex interaction of biological and psychosocial factors, where a definite aetiology has not been established. Theories suggest causative pathways between the immune and nervous systems of affected individuals with several risk factors identified in patients presenting with one or more functional syndromes. Risk factors including stress and childhood trauma are now recognised as important contributors to chronic pain conditions. Emotional, physical, and sexual abuse during childhood is considered a severe stressor having a high prevalence in functional somatic syndrome suffers. Such trauma permanently alters the biological stress response of the suffers leading to neuroexcitatory and other nerve issues associated with chronic pain in adults. Traumatic and chronic stress results in epigenetic changes in stress response genes, which ultimately leads to dysregulation of the hypothalamic-pituitary axis, the autonomic nervous system, and the immune system manifesting in a broad array of symptoms. Importantly, these systems are known to be dysregulated in patients suffering from functional somatic syndrome. Functional somatic syndromes are also highly prevalent co-morbidities of psychiatric conditions, mood disorders, and anxiety. Consequently, this review aims to provide insight into the role of the nervous system and immune system in chronic pain disorders associated with the musculoskeletal system, and central and peripheral nervous systems.

## 1. Introduction 

Functional somatic syndromes (FSS) are a group of persistent chronic pain syndromes where symptoms cannot be explained by physical or structural bodily defects or somatic disease [1]. Many terms have been used to describe FSS including hysteria, somatoform disorders, medically unexplained symptoms, and fashionable illnesses with somatic symptom disorder (SSD), conversion disorder, and illness anxiety disorder described in the Diagnostic and Statistical Manual of Mental Disorders, 5th edition [2]. FSS are considered as dysautonomia [3], i.e., a medical issue arising from the autonomic nervous system. Out of the FSS group of illnesses, the three most common include irritable bowel syndrome (IBS), fibromyalgia (FM), and chronic fatigue syndrome/myalgic encephalomyelitis (CFS/ME) with tension headaches; temporomandibular joint (TMJ) dysfunction, vulvodynia, and interstitial cystitis (IC) [4] also fall in this category. FSS are common among children and adolescents with a prevalence of 25 to 30%, being higher in females than males [5]. Additionally, symptoms can persist into adulthood impacting the physical and emotional status of the patient, with increasing age, the likeliness of recovery decreases. The theory that FSS is a subset of post-infectious syndrome with chronic symptoms emerging post-viral or bacterial infection is popular. The role of such pathogenesis in cases of FSS has not yet been proven [6]. Indeed, the aetiology of FSS has not been fully elucidated but several factors are believed to contribute to the incidence of FSS with the nervous system and musculoskeletal systems affected. Factors such as infectious disease, inflammation, mood disorders, childhood abuse, neglect, and trauma [7] contribute to the incidence of FSS. Historically, these illnesses were considered psychosomatic disorders leading to a disruption in patient–doctor relationships. It is important to recognise the overlap in FSS and mental health disorders (major depressive disorder, and anxiety disorder), where patients having a history of mental health issues are three times more likely to have an FSS [8], where a correlation or causation may be present. FSS are now commonly split into medical subspecialities (Table 1) to better establish causation and aetiological effects, where it is recognised that there is an overlap in symptoms between the syndromes and other co-morbidities [9]. The economic burden caused by these syndromes is significant with fibromyalgia itself costing twice as much as the treatment for ankylosing spondylitis and being similar to the cost of chronic lumbalgia [10]. Furthermore, these conditions are associated with absence from employment, increased demands on health care and non-health care resources, and the reliance on disability benefits due to chronic persistent symptoms [10]. The classification of symptoms as somatization where a psychopathological pathway allows the patient to convert a psychological issue (depression, anxiety, stress, neglect, abuse, etc.) into physical symptoms [11] aligns with the classification of somatic symptom disorders. Emotional or somatic awareness where patients are aware of their own somatic condition is key to psychosomatic health with disturbances resulting in autonomic homeostatic processing [12]. Persons manifesting with psychosomatic disorders display issues with emotional awareness and expression (alexithymia), which impacts several physical illnesses including FSS, gastrointestinal disorders, and chronic pain [13]. Medical science tends to adhere to Cartesian dualism separating psychiatric illness and physical symptoms with the latter being considered as real, having been caused by a physical disorder/injury. Indeed, FSS and SSD appear to have a combined biopsychosocial structure. Ongoing research, however, also indicates alternative explanations based on the interaction of biological systems such as the immune system and nervous system in the incidence of FSS allowing for some distinction between the FSS and SSD. Both the immune and nervous systems conduct vital biological functions essential for life. While initially believed to act independently, there is now a greater understanding of the interconnectedness of these two systems impacting cognition, behaviour, psychiatric illness, and neurodegenerative disease [14]. The role of immune system mediators such as cytokines and leukocytes in mental health disorders, and neurological and musculoskeletal disease is an area of much investigation. This review aims to highlight and discuss the role of the nervous system and immune system in chronic pain disorders associated with the musculoskeletal system, and central and peripheral nervous systems. 

## 2. The Nervous System Interconnects with the Immune System 

The nervous system is divided into the central nervous system (CNS) and peripheral nervous system (PNS) extending out from the CNS and having somatic and autonomic components; the latter being subdivided into the sympathetic and parasympathetic nervous systems [40]. Voluntary control of bodily functions is provided by the somatic system, which innervates skeletal muscles while the autonomic system controls visceral involuntary body functions and innervates glands [41]. The sympathetic branch of the autonomic system innervates circulatory tissue, lymph tissue, bone marrow, joints, the gastrointestinal tract (GIT), and other organs [42]. The nervous system is essential for integrating biological functions by releasing neurotransmitters and regulatory molecules, which provide physiological homeostasis [42]. Additionally, the nervous system is believed to contribute to immunity to pathogens by the modulation of cellular activity [43]. The impact of the immune system on the CNS has become of significant interest in the treatment of brain disorders via the modulation of immune components [14]. Research has identified mechanisms of neuro-immune interaction where neurons express pattern-recognition receptors (PRRs), e.g., Toll-like receptors (TLRs), and cytokine receptors, indicating that both systems are modulated by pathogen-associated molecular patterns (PAMPs), cytokines, and other immune molecules [42]. Similarly, immune cells including macrophages, dendritic cells, and T cells express receptors for neurotransmitters (acetylcholine and adrenergic), allowing for neuro-immune interaction [44]. CD4+ T cells have been shown to be key players in the onset and development of stress-related anxiety in mice [45]. Furthermore, the interactions between the enteric nervous system of the GIT and immune cells allows for response to pathogens and food molecules where enteric neurons can produce chemokines and cytokines [46]. The impact of the neuro-immune interaction in disease states including autoimmune diseases and irritable bowel syndrome (IBS) has been established. Immune cells are known to interact with nerve tissue of the GIT in patients diagnosed with IBS, FM, IC, and vulvar vestibulitis [19]. In cases of IBS, for example, there is believed to be a dysregulation of the gut–brain axis involving the CNS and PNS relating to the neurotransmitter serotonin (5-HT) [47]. Importantly, serotonin is a major neurotransmitter of the CNS and is associated with cognitive abilities, mood, sleep, and appetite. Dysregulation of serotonin is associated with many disorders of the CNS including major depressive disorder (MDD), anxiety disorders, obsessive-compulsive disorder (OCD), and psychiatric disorders including schizophrenia [17]. Serotonin also regulates the immune system and inflammation via serotonin receptors present on immune cells. This neurotransmitter attracts immune cells to sites of inflammation and regulates cell proliferation and the release of cytokines in the immune response. Additionally, immune cells including mast cells and T cells can make and release their own serotonin [48]. Catecholamines (norepinephrine, epinephrine), which are sympathetic neurotransmitters, are also regulators of immune cellular activity and inflammation via receptors present on neutrophils, macrophages, T cells, and monocytes [40]. Neurons promote the degranulation of mast cells via neuropeptides, which causes the release of histamine and other mediators of immunity, resulting in neurogenic inflammation [49]. The vagus nerve (VN), being the longest nerve in the body, connects the CNS to the organs of the body, i.e., the heart, GIT, lungs, etc. (but not the lymphoid organs), and is a major component of the parasympathetic nervous system having afferent (80%) and efferent (20%) fibres impacting immune activity [50]. The afferent fibres detect inflammation in peripheral tissues and transmit the information to the brain with the efferent fibres having anti-inflammatory activity via the cholinergic anti-inflammatory pathway [51]. The VN allows for parasympathetic nervous system control over the sympathetic and this activity is referred to as the vagal tone [52]. Activation of the efferent fibres in the CNS results in the local release of norepinephrine from sympathetic nerve terminals in the joints, which regulates the innate immune response, locally [53]. The VN stimulates the hypothalamic-pituitary–adrenal (HPA) axis regulating glucocorticoid hormone production in the adrenal glands and regulating cholinergic anti-inflammatory pathways via an inflammatory reflex [54]. Dysfunction of the HPA is also associated with autonomic and immune system dysfunction and an inability to tolerate changes in temperature [55]. Endogenic glucocorticoids play vital roles in biological processes such as metabolism, growth, and inflammation, where they have immunosuppressive activity [56]. Studies show that the VN is a key component of neuro-immune interaction where its stimulation may offer treatment for inflammatory conditions [50]. Animal studies have demonstrated the anti-inflammatory effects of stimulating the VN in the treatment of sepsis, trauma, arthritis, and endotoxemia, and such stimulation is approved for the treatment of epilepsy and depression in humans; however, its ability to regulate inflammatory conditions in humans remains unverified [51]. Certainly, such neuroimmunomodulation via activation of peripheral nerves may offer therapeutic benefits in the treatment of many diseases including inflammatory conditions and organ-specific conditions [53]. In the autoimmune disease rheumatoid arthritis (RA), for example, electrical stimulation reduced inflammatory cytokines in rat models of interleukin (IL) 1, 2, and IL6 and tumour necrosis factor (TNF) [57]. Human trials also demonstrated that stimulation of the VN reduced inflammatory symptoms in RA patients as cytokine production was inhibited [53]. Cytokines can induce FSS symptoms including fatigue, fevers, adenopathy, myalgias, arthralgias, cognitive impairment, sleep, and mood disorders [58]. Additionally, trials investigating the therapeutic effects of neuromodulation on autoimmune disease of the GIT and other disorders have demonstrated positive outcomes in some cases [59]. The immune inflammatory pathway also engages in intracellular communication in response to stress where glial cells in the brain allow for memory of past physical and psychological stressors [60]. This system allows for the fight or flight response and should return to normal levels once the danger has passed. In cases of FSS, however, this system has become dysregulated and can remain active where the body remains in a heightened state of stress response [3], explaining the relationship between trauma, adverse events, and FSS. In cases of neuropathic pain, glial cells also release neuroexcitatory molecules including proinflammatory cytokines, nerve growth factor (NGF), glutamate, reactive oxygen species (ROS), and prostaglandins, which amplify the pain response resulting in hyperalgesia and/or allodynia [52]. Studies have demonstrated that NGF increases nociception and hyperalgesia and stimulates mast cell activity, which secrete IL-17, IL-6, and TGFβ [61]. Stress is a known trigger of FSS where the neuroendocrine peptide corticotropin-releasing factor (CRF) is the key regulating biomolecule in the stress reaction of the CNS. The effects of CRF (Table 2) include regulating stress excitation, enabling endocrine, autonomic nerve, and immune interaction, and regulating behavioural response [62]. Furthermore, evidence shows that patients with CFS, IBS, musculoskeletal pain, and other FSS have a sensitized immune-inflammatory system and low-grade inflammation evidenced by increased levels of biomarkers such as C-reactive protein (CRP) compared to healthy persons [3]. This process of sensitisation of neurons or neuroinflammation in FSS patients is also referred to as neurogenic inflammation, peripheral sensitisation, visceral sensitisation, and CNS sensitization [63]. 

Importantly, the VN also offers a communication pathway between the brain and the resident GIT microbiota via the afferent fibres in the gut wall and bacterial metabolites sugars, short-chain fatty acids, and serotonin or γ-aminobutyric acid (GABA) [64]. The human microbiota has many beneficial functions where GIT dysbiosis has been linked to many disease states including autoimmunity, mood disorders, and neurodegenerative disease [65]. Studies have shown that the GIT microbiota can communicate with the brain via both endocrine (hormone) and immune (cytokine, chemokine) pathways; the VN, however, undoubtedly offers a more direct and faster route of communication [64]. Theories suggest possible links between the CNS, immune system, and the microbiota causing FSS in certain persons.

**Table 2:** Outlining the roles of varying components of both the nervous and immune systems where dysfunction is associated with FSSs.

Component	Immune Role	Neurological Role	Dysfunction	FSS and Co-Morbidities
***Cytokines***				
Interleukin	Modulate growth, differentiation, and activation of immune cells [66]	Regulate neurodevelopment, neuroinflammation, and synaptic transmission [67]	Imbalance between pro-inflammatory and anti-inflammatory cytokines [68,69]	FM, RA [68], CFS [70], IBS [71], IC [72], TMD [73], MDD, anxiety, and sleep disorders [74]
Interferon	Promote an antiviral state. Help regulate and activate immune response [75]			
Chemokines	Induce immune cell migration [69]			
***Neurotransmitters***				
Serotonin	Potent chemoattractant, modulates cytokine secretion, and cell activation/proliferation [48]	Regulates mood, appetite, sleep, nociception, motor activity, temperature, and cognitive function [76]	Alterations in the structure or expression of SERT [77].	MDD, Anxiety [17], IBS [76], FM, CFS/ME, IC, TMD [78] PHD, sleep disorders [77]
Dopamine	Regulates cytokine secretion, cell adhesion, cytotoxicity, and chemotaxis [79]	Regulates motor control, reward, and cognitive function [79]	Alterations in the levels of released DA, DA receptors, and signal transduction molecules [80]	MDD, anxiety, RA [79], FM [80], CFS/ME [81], IBS [76], TMD [82], PHD, RLS, sleep disorders [83]
GABA	Modulator of cell migration, cytokine secretion, immune cell activation, and cytotoxic responses [84]	Principal inhibitory neurotransmitter in CNS [85]	Alterations in GABA-glutamate balance [86]	FM, PHD, TMD, IBS, anxiety [86], MDD [87], CFS [88]
Glutamate	Modulator of leukocyte function, cellular adhesion and homing, dendritic cell maturation, and myeloid cell function [85]	Principal excitatory neurotransmitter in the CNS [85]		
***Hormones***				
CRF	Exerts pro-inflammatory effects; mediating mast cell activation and cytokine production [89]	Regulates stress response [90,91]	CRF hypersecretion and HPA axis hyperactivity [92]	Depression and anxiety disorders [92], IBS, endometriosis, bladder disorders [90], FM, CFS, thyroid disorders [91]

**Abbreviations:** FM—Fibromyalgia, RA—Rheumatoid arthritis, CFS/ME—Chronic Fatigue Syndrome/Myalgic Encephalomyelitis, IBS—Irritable Bowel Syndrome, IC—Interstitial Cystitis, TMD—Temporomandibular disorder, MDD—Major Depressive Disorder, SERT—Serotonin Transporter, PHD—Primary Headache Disorders, DA—Dopamine, RLS—Restless Leg Syndrome, GABA—γ-aminobutyric acid, CRF—Corticotropin-Releasing Factor, HPA—Hypothalamic-Pituitary–Adrenal.

## 3. Neuro-Immune Modulation in FSS

The immune system and the nervous system evolved to regulate bodily homeostasis, protect against invading organisms, and provide neurological communication between the CNS and organs. The immune system is mediated by organs, cells, and chemicals, which control inflammation, neutralising pathogens, and repairing damaged tissues. Inflammation can, however, become harmful resulting in chronic inflammation and autoimmune diseases [42]. Where the nervous system must allow for communication between the varying components of the body via neurotransmitters and biological regulatory molecules (Table 2). These complex evolutionary systems maintain optimal bodily conditions allowing for survival and growth in the environment of the organism. In cases of functional somatic syndrome, however, there is a disconnect between these two systems resulting in chronic pain conditions, neuropathic pain, and functional issues of the digestive tract amongst other issues (Table 2). Central sensitization is associated with neuroinflammation via activation of inflammatory immune cells (macrophages, mast cells, fibroblasts, etc.) and inflammatory molecules (cytokine, chemokines). As these immune mediators interact with nociceptors on the CNS and PNS changes occur in pain pathways (excitability, conductivity, and transmission) leading to increasing pain or amplification of pain in the patient [93]. Studies demonstrate that fibroblasts secrete cytokines and chemokines that can induce peripheral neuron sensitization causing peripheral neuron hypersensitivity and chronic pain [94]. Neuropathic pain occurs from a primary lesion or dysfunction of the central and peripheral nervous systems [95] and is usually chronic and persistent with limited treatment options. 

### 3.1. Fibromyalgia

Fibromyalgia is a syndrome presenting with widespread musculoskeletal pain, muscular dysfunction, hyperalgesia, allodynia, sleep disorders, cognitive issues, fatigue, and mood disorders with a prevalence of 5% predominantly affecting females [96]. Typically, FM patients have a reduced ability to tolerate pain and extreme variations in heat and cold temperatures [97]. Studies indicate that FM pain is comparable to the pain of RA, which is dysesthetic, such as painful burning, prickling, aching, or paroxysmal and temperature sensitive [98]. Establishing the role of the immune system in FM is an ongoing area of research where studies have shown altered levels of certain cytokines in FM patients, including IFN-γ, IL-5, IL-6, IL-8, and anti-inflammatory IL-10 upon activation of peripheral blood mononuclear cells (PBMCs) [99]. Importantly, the pro-inflammatory pleiotropic cytokine IL-6 is associated with neoplasia and autoimmune diseases [71]. Studies also report increased levels of pro-inflammatory cytokines post PBMC stimulation [96] leading to chronic low-grade inflammation. Adipose tissue also acts as a source of such pro-inflammatory cytokines, where increased nociceptive activity is also present in obese individuals [100]. The constant low-grade inflammation present in FSS patients sensitizes the neurons involved in sensing pain, transmitting pain signals, and representing pain in the CNS, making them more excitable. Excitable neurons then signal the perception of pain to the CNS more easily with less irritation than the non-excitable neurons of non-FM patients [3]. There is an evident alteration in the brain in FM patients with a default present between the pain inhibitory centres and the insular cortex and higher levels of glutamate compared to healthy persons [63]. Glutamate is the primary neurotransmitter released by vagus nerve sensory neurons [40]. Neurogenic inflammation also results from pro-inflammatory cytokines activating innate and adaptive immunity, which are secreted by afferent nerves in local tissues sending pain signals to the CNS. Central neuroinflammation and elevated neuropeptides (substance P), brain-derived neurotrophic factor, and Nerve Growth Factor (NGF) have been identified at elevated levels in the spinal fluid of FM patients [97]. The substance P neuropeptide engages in the pathophysiology of pain where NGF is associated with hyperalgesia and detection of painful stimuli (nociception). Studies also show an increased presence of mast cells in the skin and blood vessels of FM patients where secretory granules can release inflammatory and neuro-sensitising molecules including bradykinin, histamine, TNF, and tryptase [97]. The communication between mast cells and microglia via histamine and tryptase can also release inflammatory cytokines causing an innate immune reaction in the brain contributing to brain inflammation and brain disorders [101]. Microglia cells are macrophages that survey and clear pathogens in the CNS. Histamine and tryptase can change nociceptive visceral sensory nerve function resulting in nerve stimulation and hypersensitivity [102]. FM pain involves neuroinflammatory processes triggered by mast cells and microglia immune cells via the secretion of cytokines in the CNS. FM patients also have increased levels of the inflammatory chemokines CCL17, CXCL9, CCL22, CXCL11, and CCL11, which attract innate and immune cells [45]. Studies also report increased activity of the sympathetic nervous system in FM patients, which can moderate the peripheral nociceptor neurons indicating peripheral neuroinflammation [63]. Peripheral nociceptors involved in pathological pain express cytokine and chemokine receptors suggesting neuro activity is directly activated by the immune system resulting in excitability, termed peripheral sensitization [93]. There is evidence of abnormalities in the small nerve fibres of approximately 50% of FM patients where nerves are thinly myelinated or unmyelinated (small fibre polyneuropathy) and the average axon diameter is also reduced [103], highlighting the importance of the peripheral nervous system in FM. Alterations of C-fibres and Schwann cells are also evident [104]. The alterations in Schwann cells, nerve fibre density, and the diameter of the axons in FM patients have been observed in many chronic pain conditions [105]. Schwann cells function to detect and respond to nerve injury by altering their phenotype, proliferating, and releasing growth factors, immune mediators, i.e., cytokines, chemokines, and other molecules that interact with nociceptive neurons [95], impacting on neuropathic pain. C-fibres of primary afferent nerves are activated by stimuli and result in the sensation of pain [98]. Certain FM patients appear to suffer from the spontaneous activity of peripheral nerves and sensitisation of C-fibre nociceptors [98]. Additional observations in FM patients include dopamine dysregulation [17], alteration of endogenous cerebral opioids, neuroendocrine factors, oxidative stress, genetics, and psychosocial factors [100]. The HPA contains stress-induced neurotransmitter and neuroendocrine response systems, which are also believed to be involved in FM. Increased levels of free radicals are present in FM patients who also have a decreased antioxidant ability; free radicals excessively impact the CNS due to their high lipid content [100]. 

### 3.2. Irritable Bowel Syndrome

IBS is considered a functional gastrointestinal condition with intermittent abdominal pain and cramping associated with bowel movements. Visceral hypersensitivity is also considered a symptom of IBS where patients have a lower tolerance for and increased sensitivity to pain [106]. While recognised as an FSS, IBS also falls into the category of disorders of gut–brain interactions (DGBIs), or functional gastrointestinal disorders (FGIDs) [49]. IBS is generally divided into four types depending on the bowel manifestations including IBS diarrhoea (IBS-D), IBS constipation (IBS-C), IBS mixed (IBS-M), and unclassified IBS [102]. IBS has a prevalence rate of 10–25%; the pathogenesis of disease, however, remains unclear. It is generally accepted that dysregulation of the autonomic nervous system (dysautonomia), the HPA, increased sensitivity to pain, and gut dysbiosis contribute to IBS [62]. The role of the immune system in IBS pathology remains an area of much interest. Studies highlight the role of the immune system in IBS as symptoms often manifest post-gastrointestinal infection, and there is a greater prevalence in inflammatory bowel disease (IBD) patients in remission [19]. Ongoing research also demonstrates the presence of low-grade inflammation, innate immune dysfunction, and cytokine imbalance in IBS patients [102]. TNF-α, IL-1β, and IL-17 serum levels have been associated with abdominal pain and severity of symptoms in IBS patients with IL-6 and IL-8 elevated in some cohorts [71]. Decreased levels of the anti-inflammatory cytokine IL-10 are also present. The pro-inflammatory IL-1β can induce an inflammatory reaction, affect smooth muscle, and damage the mucosal barrier of the GIT [62]. The GIT also has an abundance of resident eosinophils and mast cells, which are found near nerve tissue allowing for communication between the nervous and immune systems [107]. These intestinal eosinophils secrete chemokines, cytokines, substance P, CRF, and other peptides, which may play a role in GIT disease states including IBS [108]. Studies demonstrate elevated levels of CRF in intestinal eosinophils correlate with the severity of symptoms in IBS-D patients [108]. Interestingly, 70% of mast cells in the GIT are in direct contact with nerve cells and function to regulate intestinal permeability, peristalsis, nociception, and innate and adaptive immunity amongst other functions [109]. Mast cells are also activated to release chemical mediators (histamine, serotonin, cytokines, chemokines, and tryptase) by stress-induced neural stimulation in IBS patients [107]. Enteric mast cells are triggered by neuropeptides such as vasoactive intestinal peptides to release histamine and other immune mediators resulting in neurogenic inflammation in IBS patients [49]. The role of the enteric nervous system in the pathophysiology of IBS is also not fully elucidated. The enteric nervous system consists of millions of neurons and glial cells (present in ganglia) and is sub-divided into the submucosal plexus and the myenteric plexus regulating muscular, neuro-hormonal, and secretory systems of the GIT, allowing for digestive action [110]. The findings of Ostertag, 2015, show altered neuron activity in the submucosal plexus of patients presenting with IBS, which were stimulated by immune mediators [111]. The enteric nervous system influences the intestinal epithelia, endocrine and immune systems, and the gut microbiota [43]. The enteric system has a central role in regulating gastrointestinal activity and physiology where alterations in this complex nervous system result in imbalanced GIT homeostasis, and gastrointestinal and extra-gastrointestinal disease states [49]. The communication pathway between the gut microbes and the VN is also implicated in GIT disorders where dysbiosis impacts both IBS and IBD in patients [59]. The neuroactive biologics secreted by the resident microbes (GABA, serotonin, dopamine, and acetylcholine) influence the enteric nervous system and the VN to influence the brain and peripheral organs [112]. Dysbiosis of the GIT is associated with leaky gut, visceral hypersensitivity, immune activation, inflammation, mood disorders, and chronic fatigue in IBS patients [113]. 

### 3.3. Chronic Fatigue Syndrome/Myalgic Encephalomyelitis

Chronic fatigue syndrome/myalgic encephalomyelitis is a chronic condition of debilitating fatigue without relief after rest, musculoskeletal pain, sleep disturbances, neuroinflammation, and cognitive impairment [114] in the absence of a patho-physiologic cause. CFS is classified by the WHO as a disease of the nervous system [115]. Viral pathogenesis is the most common trigger for CFS; stress, trauma, and childhood adverse events, however, are also risk factors impacting the severity of illness [3]. Pathogens can trigger glial cells to release neuroexcitatory molecules that act on the VN causing a pain response, which can lead to hyperalgesia and allodynia, which supports the theory of post-infection development of CFS [115]. Stress as a causative factor of CFS relates to the HPA where stress leads to a loss of cortisol production impacting immune function. A decrease in cortisol (anti-inflammatory) and adrenocorticotropic hormone (ACTH) production causes serotonin and CRH dysfunction in CFS patients [115]. CFS shares many features with FM with differentiating factors of predominant fatigue and post-exertional illness with evidence of HPA dysfunction [114]. Additional symptoms include slight fever, pharyngodynia, laterocervical, or axillary lymphadenopathy, generalized myasthenia, myalgia, headache, sleep disorders, and neuropsychological disorders such as photophobia, amnesia, irritability, mental confusion, and difficulty concentrating [115]. Symptoms vary from mild to severe with approximately 75% of patients unable to work and 25% housebound and even bedbound [116]. The aetiology of CFS remains undetermined; however, neurologic, immunologic, and autonomic mechanisms are considered important factors [116]. Additionally, energy metabolism and the mitochondria appear important in the aetiology of CFS. The mitochondria are the energy-producing powerhouse of cells where ATP is manufactured, where impaired ATP production and energy metabolism may cause the chronic fatigue featured in CFS [117]. Indeed, studies have shown a deficit in the energy-producing pathways involving simple sugars, proteins, and fatty acids [58]. Dysfunction of the autonomic nervous system is characteristic of CFS including orthostatic hypotonia, GIT disturbances, and orthostatic tachycardia syndrome [118]. Certain CFS patients present with antibodies towards muscarinic, acetylcholine and beta (β) adrenergic receptors, and cerebral cytokine dysregulation [119]. Neuro activity via β-adrenergic receptors, specifically β2-adrenergic receptors, modulates anti-inflammatory activity [40]. Studies show additional characteristics of reduced cerebral blood flow and altered sympathetic and para-sympathetic signalling in the brain stem [119]. Dysfunction of the VN is also present in CFS patients where the vagal tone is abnormal, leading to changes in heart rate and respiration [52]. The neuro-immune or inflammatory reflex mediated by the VN appears important in the development of CFS. Immune system dysfunction in CFS patients relates to the presence of autoantibodies, impaired natural killer cells, excess cytotoxic T cells, and higher levels of pro-inflammatory cytokines [58].

### 3.4. Co-Morbidities of Patients with FSS

Phycological disorders are prominent in FSS patients, with 25% of IBS and 51% of FM patients being diagnosed with depression or anxiety in the 5 years pre-diagnosis [6]. Studies demonstrate an overlap in IBS and mood disorders in FM patients having similar neurobiological alterations [4]. As with FSS, alterations of the CRF and HPA axis are present in patients with anxiety and depression [106]. FM is also associated with migraine, TMJ dysfunction, pelvic pain, complex regional pain syndrome, restless legs syndrome, IBS, interstitial cystitis, hypotension, and hypersensitivities [63]. Neuroinflammation in FM patients is believed to be triggered by stress, pain, dysbiosis of the gut, and vitamin D deficiency, which are also predisposing factors for autoimmune diseases [114]. Furthermore, there is a higher prevalence of autoimmune disease in FM patients including RA, lupus erythematosus, Sjogren’s syndrome, IBD, and Type 1 diabetes (T1D), amongst others. Increased levels of mast cells and their mediators in close proximity to enteric neurons is a feature of both IBD and IBS [49] where there is also an overlap of IBS in IBD patients. Autoantibodies anti-polymer antibody (APA), anti-68/48 kDa, and anti-45 kDa have been detected in FM, CFS, and patients presenting with psychiatric disorders [100], which may serve as biomarkers in certain cases. The higher prevalence of autoimmune diseases and FSS in females suggests some risk factors relating to the sex hormones in both conditions. Sex hormones can also trigger masts cell activity contributing to innate immunity, autoimmunity, and inflammation where oestrogen receptors have been identified on rodent mast cells [93]. 

Cyclic vomiting syndrome is a chronic FGID with symptoms of nausea, vomiting (emesis), and intestinal pain; gastroparesis (delayed gastric emptying) and migraine are also co-morbidities of IBS [120]. Some hypothesise that CVS is due to psychological or infectious triggers causing alterations or neuroendocrine dysfunction of the gut–brain pathway and activation of the CRF system [121]. Activation of CRF can result in emesis and gastroparesis by stimulation of the inhibitory motor nerves in the dorsal motor nucleus of the VN [122]. The autonomic nervous system has a major role in both emesis and nausea via the visceral afferent fibres of the VN in the GIT [123]. Co-morbidities of CFS/ME include FM, IBS, chemical sensitivity, thyroiditis, Raynaud’s syndrome, interstitial cystitis, TMJ syndrome, and headaches [26]. CFS patients may also be more prone to acute viral infections due to immune dysfunction [58]. Importantly, the prevalence of FSS may increase in a post-pandemic era where studies show that patients suffering from long-covid post-COVID-19 infection meet the criteria for CFS diagnosis [116]. 

Treatment options for FSS patients remain under question, with exercise, diet, cognitive behavioural therapy (CBT), and sleep recommended for preventing an FSS flare-up. CBT appears most beneficial in the treatment of FSS [2]. Regular exercise improves sleep, fatigue, and physical function in FSS patients having similar benefits to CBT. Treatments aimed at preventing nociceptive neurotransmitters in the CNS have been trialled for FM patients. Currently, pregabalin an active pharmaceutical ingredient (API), which decreases neuronal activity, and duloxetine and milnacipran (serotonin and noradrenaline reuptake inhibitors) are approved for the treatment of FM [4]. Studies reporting the efficacy of antidepressant drug therapy for the treatment of FSS are lacking. Indeed, some studies report a lack of efficacy of antidepressant therapy in FSS patients [2]. Typically, non-steroidal anti-inflammatory therapeutics are ineffective in FSS. The use of CRF antagonists may offer some advantages such as decreasing visceral hypersensitivity and colonic motility [106]. The use of chromones (mast cell stabilizers) in patients with IBS is also an area of potential therapy [19]. 

## 4. Conclusions

The nervous system and the immune system provide essential biological roles where a complex interaction between both systems is essential in maintaining health and wellbeing. Dysregulation of these systems, however, can result in numerous chronic pain conditions including functional somatic syndrome. In chronic pain conditions, the pain system of the patient is triggered by numerous mechanisms including permanently activated nociceptive nerves (visceral sensitivity, neurogenic inflammation), CNS nerve cells activation (central sensitisation, neuroinflammation), and where the perception of pain in the brain remains active (neuroinflammation, central sensitisation). Undoubtedly, FSS are multifaceted syndromes with varying risk factors, having a negative impact on many bodily systems. Predisposing factors including genetics, infectious disease, mood disorders, autoimmunity, and alterations in central and visceral nerve sensitivity are all contributing factors. Indeed, some hypothesise that FSSs FM and CFS are manifestations of psycho-neuro-endocrine–immune disorders. Importantly, the contribution of childhood trauma (emotional, sexual, and physical abuse) appears significant in the manifestation of FSS, which endures into adulthood as it permanently alters the body’s stress-response systems and neuroexcitatory pathways. Traumatic and chronic stress is believed to be a risk factor for FSS as long-term stress and trauma can result in dysregulation of the HPA, autonomic nervous system, and immune system. The neural pathways regulating the processing of pain are closely linked to the autonomic nervous system. Studies report decreased parasympathetic activity and sympathetic reactivity to physical and stress triggers in FSS patients. There is an urgent need to identify biomarkers for FSS, perhaps pro-inflammatory cytokines, neuropeptides, glutamate, and auto-antibodies can indicate the presence of FSS in conjunction with patient symptoms. While patients with FSS pose a unique challenge to clinical staff, issues arise in relation to the classification of FSS as somatic or mental disorders causing patient–doctor frustration where patients often feel ignored or dismissed. The role of alexithymia in patients with FSS, and psychosomatic disorders is also worthy of exploration as alexithymia is known to disrupt autonomic homeostatic functioning. Advances in immunology, neuroscience, and neuroimmune pathways have undoubtedly furthered our understanding of the molecular mechanism involved in chronic pain conditions. A better understanding of neuro-immune communication and regulation will ultimately lead to better therapeutic options for long-suffering patients of FSS and neurological, inflammatory, and autoimmune diseases. 

## Figures and Tables

**Table 1:** Outlining FSSs, symptoms, diagnosis, common co-morbidities, or clinical overlap.

Functional Somatic Syndrome	Symptoms	Diagnosis	Medical Subspecialty	Clinical Overlap
Fibromyalgia	Chronic widespread muscular pain, hyperalgesia, allodynia, sleep disturbances, physical exhaustion, GI problems, and cognitive difficulties [15]	18 tender points, chronic pain, widespread symptoms [16]	Rheumatology	MDD [17], anxiety, IBS [4], rheumatism [15], IC [16], TMD [18]
IBS	Changes in bowel habit, somatization [19]	Pain relief after defecation, bloating and distention, cramping, nausea [20]	Gastroenterology	Functional dyspepsia [21]
CFS/ME	Severe and disabling fatigue, sleep disruption, unrefreshing sleep, PEM, tender lymph nodes, palpitations, multifocal pain, hyperalgesia, GI problems, and cognitive dysfunction [22]	Longstanding unexplained fatigue, PEM, chronic myalgia and cognitive impairment [23]	Infectious disease [9]	FM, POTS [24], IBS [25], TMD, IC, Raynaud’s disease, thyroiditis [26], depression, mood and anxiety disorders [27]
Tension headaches	Chronic dull, aching, pressure-like head pain [28]	Bilateral, tightening and oppressive headache, mild to moderate pain [29]	Neurology	TMD, FM, sleep disturbances, anxiety, and depression [30]
TMJ dysfunction	Chronic pain in masticatory muscles and TMJs, headache, ear pain, disturbances in jaw movements, facial pain, neck and shoulder tenderness [31]	Jaw movement limitation, irregular TMJ noises (clicking, popping, grating, crepitus), diagnostic imaging (radiography, CT scan, MRI) [32]	Musculoskeletal	TTH, cluster headache, migraine [33]
Vulvodynia	Burning, stinging, or throbbing vulvar pain, dyspareunia [34]	Chronic idiopathic vulvar pain, cotton swab test (check for trigger points) [35]	Gynaecology	FM, IBS, CFS/ME, IC, endometriosis [36]
Interstitial cystitis	Bladder and pelvic pain, frequent, urgent, and painful urination, dyspareunia [37]	Reoccurring idiopathic pain in bladder, pelvis, and perineal area. Pressure/discomfort such as bladder fills, relief after urination. Cystoscopy [38]	Gynaecology, urogynaecology	IBS, FM, CFS/ME, endometriosis, vulvodynia, Sjogren’s syndrome, anxiety disorders [39]

**Abbreviations:** MDD—Major Depressive Disorder, IBS—Irritable Bowel Syndrome, TMD—Temporomandibular Joint Disorder, IC—interstitial cystitis, CFS/ME—Chronic Fatigue Syndrome/Myalgic Encephalomyelitis, PEM—Post-Exertional Malaise, FM—Fibromyalgia, POTS—Post-Orthostatic Tachycardia Syndrome, TMJ—Temporomandibular Joint, TTH—Tension-type headache.

## Data Availability

Not applicable.
